# Dry season occurrence of *Anopheles* mosquitoes and implications in Jabi Tehnan District, West Gojjam Zone, Ethiopia

**DOI:** 10.1186/s12936-018-2599-4

**Published:** 2018-11-29

**Authors:** Abebe Animut, Yohannes Negash

**Affiliations:** Aklilu Lemma Institute of Pathobiology, Addis Ababa University, P. O. Box 1176, Addis Ababa, Ethiopia

## Abstract

**Background:**

Generating evidence on the dry season occurrence of the larval and adult stage**s** of *Anopheles* mosquitoes helps to design effective malaria vector control strategy as the populations of the vectors is expected to be low.

**Methods:**

Larval and adult stages of *Anopheles* were surveyed during dry seasons in Mender Meter, Jiga Yehlmidar and Wongie Berkegn villages, Jabi Tehnan District, West Gojjam Zone, Ethiopia. Larvae were surveyed (along the available surface water collections), sampled, identified into genus, counted and late instars of the genus *Anopheles* identified into species. Indoor-resting adult mosquitoes were collected using insecticide aerosol spray, processed and identified into species. Data was analysed using SPSS version 20.0 to determine frequencies, mean differences and associations.

**Results:**

A total of 3127 *Anopheles* larvae were collected among which most (91.7%; 2869/3127) were from streams followed by ponds (4.3%; 136/3127) and swamps (3.9%; 122/3127). *Anopheles gambiae sensu lato* was the most prevalent (84.9%; 921/1085) followed by *Anopheles cinereus* (7.0%; 76/1085), *Anopheles chrysti* (3.7%; 40/1085), *Anopheles demeilloni* (2.8%; 30/1085) and *Anopheles rhodesiensis* (1.6%; 18/1085). The mean number (mean = 15.3) of *An. gambiae* from Jiga Yehlmidar was significantly (p = 0.024) higher than the corresponding number (mean = 3.2) from Mender Meter. The mean number (mean = 36.3) of *An. gambiae* larvae in April 2017 was significantly (p = 0.001) higher than the number (mean = 4.0) in December 2013 and the number (mean = 2.6) in March 2013. A total of 1324 adult *Anopheles* were collected of which the highest proportion (79.1%; 1048/1324) was *An. gambiae*, followed by *An. chrysti* (11.7%; 155/1324), *An. demeilloni* (6%; 80/1324), *An. cinereus* (2.6%; 35/1324) and *Anopheles coustani* (0.5%; 6/1324). The highest proportion (54.3%; 569/1048) of the *An. gambiae* was collected from Wongie Berkegn followed by Jiga Yehlmidar (23.6%; 247/1048) and Mender Meter (22.1%; 232/1048). The mean number (mean = 7.8) of adult *An. gambiae* caught in Wongie Berkegn was significantly (p = 0.018) higher compared to the number (mean = 3.0) in Mender Meter. No significant difference was observed in the mean number of adult *An. gambiae* between the seasons.

**Conclusion and implication:**

Streams were prolific breeding habitats of *Anopheles* mosquitoes followed by ponds and swamps in the dry seasons. In addition, a high population of indoor resting *An. gambiae* was caught from indoors. This implies the need for a strengthened vector control during dry seasons using breeding habitat management and improved housing in addition to the existing insecticide (LLINs and IRS) based interventions in Jabi Tehnan District, West Gojjam Zone, Ethiopia.

## Background

Malaria vectors undergo egg, larval, pupal and adult stages in their life time. Their egg, larval and pupal stages are limited to water bodies and have very small spatial dispersion. In dry seasons, the number and size of larval habitats is generally believed to reduce significantly and contribute to a low population of malaria-transmitting adult *Anopheles* [1–3]. The narrow spatial dispersion and low population size of the immature vectors make them more amenable to environmental management [4, 5]. Thus, larval habitat management can best be implemented in dry seasons in the fight against malaria vectors. Even so, *Anopheles* species exploit a variety of breeding habitats that vary considerably in size, altitude, vegetation cover and topography [1, 6, 7]. In a narrow topographic area, breeding habitats can have enormous variability in their anopheline productivity [7–9]. The majority of vectors may emerge from prolific habitats, which could account only for a small proportion of the habitats. Thus, effective larval interventions can be achieved through targeting productive habitats during dry seasons [6].

After emergence from pupal stage, malaria-transmitting adult female *Anopheles* undertake mating with their male counterparts and find human blood meal to nourish their eggs. They imbibe blood mainly during night hours inside residential houses and rest in door until their blood meal is digested and eggs are matured. Accordingly, insecticide-impregnated mosquito nets and residual insecticide sprays are employed inside houses to prevent and kill indoor biting and indoor resting vectors, respectively. In dry seasons, the adult vector population may reduce significantly owing to the reduction in breeding habitats [2, 3, 10]. Even so, some may survive due to factors, such as behavioural adaptation and insecticide resistance [4, 11]. They may prefer some housing conditions over others [12] and show a pattern of clustering in which case a great majority of them could occur inside smaller proportion of the houses built near their breeding habitats.

The relatively small populations of aquatic and adult stages in dry seasons could explode to very large populations during and immediately after wet seasons. Wet seasons usually make vector control interventions more difficult and expensive compared to the dry seasons in areas where dryness is associated with rare and highly limited breeding habitats. In such areas, control on larval and adult vector populations during the dry seasons can cause a significant reduction on the existing vector populations and could delay or reduce vector population explosion during and following the rains. Dry season occurrence of malaria vectors was assessed through surveys of immature and adult *Anopheles* in Jabi Tehnan District, West Gojjam Zone, Ethiopia.

## Methods

### Study area

The study was undertaken in Mender Meter [1912 m above sea level (m.a.s.l.)], Jiga Yehlmidar (1818 m.a.s.l.), and Wongie Berkegn (1786 m.a.s.l.) villages of Jabi Tehnan District, West Gojjam Zone, Ethiopia where malaria is endemic [13, 14]. These villages were also used to study the distribution and use of vector control tools [13] and current malaria prevalence [14]. In each village, available surface water collections (streams, swamps, ponds, irrigation channels) and water wells were surveyed for *Anopheles* larvae. The streams cross pasture and farm lands while the swamps and ponds were located in the pasture lands. In each village, a minimum of 10 randomly selected houses, located within a radius of 1 km from the nearest stream/breeding habitat were surveyed during each season.

### Survey of malaria vector breeding habitats

Larval and adult stages *Anopheles* were surveyed during three dry seasons (March 15–20, 2013, December 19–21, 2013 and April 17–20, 2017). Potential mosquito breeding habitats were first inspected for the presence of mosquito larvae visually and positive habitats were sampled with a 350 ml capacity soup ladle. The water was left to settle until larvae came to the surface after each subsequent dipping. Ten dips were made from each potential breeding habitat, each dip having a volume of about 80% of the liquid holding capacity of the ladle to prevent spill off. The larvae from the dipper were transferred to white enamel trays for further categorization into genus and instars. *Anopheles* larvae were categorized as first instar, second instar, third instar and/or fourth instar. Early *Anopheles* larval instars (first and second) were discarded after counting. The late *Anopheles* larval instars were preserved in 70% alcohol after they were killed in hot water (ca. 60 °C), mounted in gum-chloral mountant on microscopic slides and morphologically identified to species under microscope [15, 16]. Larval sampling was made by the first author (AA) from 11:00 am to 2:00 pm.

### House survey for indoor-resting *Anopheles*

A survey of indoor-resting *Anopheles* was made during three dry seasons (March 16–18, 2013, December 20, 2013–January 2, 2014 and April 16–18, 2017) using insecticide aerosol spray. The insecticide aerosol, named Roach Killer, contained Fenithrothion, Cypermethrin and Bioallethrin active ingredients. The spray was made in the morning (7:00 am to 8:30 am). Before spraying occupants, domestic animals, utensils used for food, food, drinking water and clothes were taken out of houses. The major house apertures were covered with clothes, and the available floor space was covered by white plastic sheets. Spraying was made according to the manufacturer’s instruction and collectors waited outside for about 10 min. The sheet was then carefully taken out of the house and knocked down mosquitoes were collected using forceps [17, 18].

Female anophelines of all catches were counted and identified morphologically to species under dissecting microscope [16, 19]. Condition of each spray house including altitude, presence of open eave, door fitness, presence of hole on wall/roof, presence of window, type of roof (thatched/corrugated iron), number of people slept inside the house the previous night, number of domestic animals (cattle, sheep, goat, horse, donkey, chicken) tethered inside the house the previous night, presence of bed net, number of people who slept under bed net the previous night, and house distance from the nearest breeding site were recorded [12].

### Statistical analysis

Data were entered and analysed using IBM SPSS Statistics Version 20.0. Frequencies of aquatic stages and indoor resting *Anopheles* were determined. One-Way ANOVA analysis with Tukey Post-Hoc tests was used to compare the differences in the mean number of larval and indoor-resting adult stages of *Anopheles gambiae sensu lato* (*An. gambiae* thereafter) between seasons and villages after log(x + 1) transformed. Association of the variables of the major housing conditions with the transformed *An. gambiae* was analysed using binary and multiple regression in order to determine the strong predictor variable(s). Variables with p values < 0.05 were considered significant.

## Results

### *Anopheles* larvae occurrence and distribution

A total of 3127 *Anopheles* larvae were collected from the available surface water collections from Mender Meter, Jiga Yehlmidar and Wongie Berkegn villages in March 2013, December 2013 and April 2017 (Table 1). The highest number (n = 2077) of *Anopheles* larvae catches was made in Wongie Berkegn followed by Jiga Yehlmidar (n = 655), and Mender Meter (n = 395). Density of larvae was highest in April, 2017 (n = 2011) followed by March 2013 (n = 612) and December 2013 (n = 504). The streams contributed to the great majority of the larvae caught (91.7%; 2869/3127) followed by the ponds (4.3%; 136/3127) and swamps (3.9%; 122/3127). From among the total *Anopheles* larvae, 62.6% (1958/3127) were early instars and 37.4% (1169/3127) were late instars.

**Table 1:** Occurrence of *Anopheles* larvae by village, season and habitat type in Jabi Tehnan District, West Gojjam Zone, Ethiopia, March 2013–April 2017

Village	Season	Habitat	1st instar	2nd instar	3rd instar	4th instar	Total	Season total	Village total
Mender Meter	Mar 2013	Stream	0	0	0	0	0	0	395
		Pond	0	0	0	0	0		
	Dec 2013	Stream	45	70	54	34	203	311	
		Pond	39	25	23	21	108		
	Apr 2017	Stream	33	19	21	11	84	84	
Jiga Yehlmidar	Mar 2013	Stream	35	24	26	13	98	98	655
	Dec 2013	Stream	9	7	12	2	30	94	
		Pond	1	8	16	3	28		
		Swamp	6	1	13	16	36		
	Apr 2017	Stream	156	66	94	61	377	463	
		Swamp	26	11	27	22	86		
Wongie Berkegn	Mar 2013	Stream	227	201	58	28	514	514	2077
	Dec 2013	Stream	80	12	6	1	99	99	
	Apr 2017	Stream	470	387	357	250	1464	1464	
Total			1127	831	707	462	3127	3127	3127

Among a total of 1169 late instar *Anopheles* larvae, 92.8% (1085/1219) were morphologically identified into species (Table 2). *Anopheles gambiae* was the most prevalent (84.9%; 921/1085) followed by *Anopheles cinereus* (7.0%; 76/1085), *Anopheles chrysti* (3.7%; 40/1085), *Anopheles demeilloni* (2.8%; 30/1085) and *Anopheles rhodesiensis* (1.6%; 18/1085). Density of *An. gambiae* was highest in Wongie Berkegn (72.1%; 664/921) followed by Jiga Yehlmidar (23.6%; 217/921) and Mender Meter (4.3%; 40/921). The mean number (mean = 15.5) of *An. gambiae* from Wongie Berkgen was significantly (p = 0.011) higher than the corresponding number (mean = 3.2) from Mender Meter. The mean number (mean = 15.3) of *An. gambiae* from Jiga Yehlmidar was significantly (p = 0.024) higher than the corresponding number (mean = 3.2) from Mender Meter. No significant difference was observed in the mean numbers of *An. gambiae* collections between Jiga Yehlmidar and Wongie Berkegn. The mean number (mean = 36.3) of *An. gambiae* larvae in April 2017 was significantly (p = 0.001) higher than the number (mean = 4.0) in December 2013 and the number (mean = 2.6) in March 2013. Similarly, the mean number of catches in March 2013 was significantly (p < 0.05) different from the number in December 2013. *An. gambiae*, the most prevalent mosquito in the area, was found to breed along streams, swamps and ponds. Water wells and irrigation channels were positive for culicine larvae but negative for *Anopheles* larvae. Streams were the most productive habitats of *An. gambiae* (91.7%; 845/921) followed by swamps (6.8%; 63/921) and ponds (1.5%; 14/921).

**Table 2:** Species distribution of *Anopheles* larvae by village and habitat type in Jabi Tehinan District, West Gojjam Zone, Ethiopia, March 2013–April 2017

Season	Species	Mender Meter		Jiga Yehlmidar			Wongie Berkegn	Total
		Stream	Pond	Stream	Pond	Swamp	Stream	
Mar 2013	*An. gambiae*	0	0	5	0	0	72	77
	*An. cinereus*	0	0	0	0	0	6	6
	*An. chrysti*	0	0	0	0	0	1	1
	*An. demeilloni*	2	0	0	0	0	2	4
	*An. rhodesiensis*	0	0	0	0	0	1	1
Dec 2013	*An. gambiae*	12	1	13	12	28	7	73
	*An. cinereus*	36	3	0	3	0	0	42
	*An. demeilloni*	0	2	0	0	0	0	2
	*An. chrysti*	0	0	0	6	0	0	6
	*An. rhodesiensis*	15	0	0	1	0	0	16
Apr 2017	*An. gambiae*	27	0	124	0	35	585	771
	*An. cinereus*	2	0	13	0	6	7	28
	*An. chrysti*	1	0	12	0	3	17	33
	*An. demeilloni*	9	0	5	0	5	5	24
	*An. rhodesiensis*	0	0	0	0	0	1	1
Total		104	6	172	22	77	704	1085

### In-door occurrence of adult *Anopheles* mosquitoes and their determinants

A total of 1324 adult *Anopheles* mosquitoes were collected from indoors by aerosol spray sheet collection (ASC) (Table 3). Among these, the highest proportion (79.1%; 1048/1324) was *An. gambiae*, followed by *An. chrysti* (11.7%; 155/1324), *An. demeilloni* (6%; 80/1324), *An. cinereus* (2.6%; 35/1324) and *Anopheles coustani* (0.5%; 6/1324). The highest proportion (54.3%; 569/1048) of the *An. gambiae* was collected from Wongie Berkegn followed by Jiga Yehlmidar (23.6%; 247/1048) and Mender Meter (22.1%; 232/1048). The mean number (mean = 7.8) of adult *An. gambiae* caught in Wongie Berkegn was significantly (p = 0.018) higher compared to the number (mean = 3.0) in Mender Meter. The difference in the mean number of indoor-resting *An. gambiae* between Jiga Yehlmidar and Wongie Berkegn was not statistically significant. Density of *An. chrysti* was also observed to have a similar trend with that of *An. gambiae*, but *An. demeilloni* was found to decrease with a decrease in altitude.

**Table 3:** Occurrence of adult *Anopheles* species by season and village in Jabi Tehinan District, West Gojjam Zone, Ethiopia, March 2013–April 2017

Season	Species	Mender Meter	Jiga Yehlmidar	Wongie Berkegn	Total
		N	N	N	
Mar 2013	*An. gambiae*	6	107	191	304
	*An. chrysti*	7	28	3	38
	*An. demeilloni*	1	7	1	9
	*An. cinereus*	0	10	0	10
	*An. coustani*	0	2	0	2
Dec 2013–Jan 2014	*An. gambiae*	109	64	177	350
	*An. chrysti*	2	8	36	46
	*An. demeilloni*	33	8	6	47
	*An. cinereus*	2	5	0	7
	*An. coustani*	0	0	1	1
Apr 2017	*An. gambiae*	117	76	201	394
	*An. chrysti*	0	15	56	71
	*An. demeilloni*	0	15	9	24
	*An. cinereus*	0	18	0	18
	*An. coustani*	0	0	3	3
Total		277	363	684	1324

Adult *An. gambiae* (n = 394) caught in April 2017 was the highest followed by December 2013–January 2014 (n = 350) and March 2013 (n = 304). However, no significant difference was observed in the mean number of the adult *An. gambiae* between the seasons. The indoor occurrence of *An. gambiae* was similar among all the housing conditions as analysed by the regression statistics. None of the variables of the housing conditions was strongly associated with the density of indoor resting *An. gambiae* and also none was found to be a strong predictor of indoor resting *An. gambiae* in the villages.

## Discussion

Streams were the most prolific breeding habitats of *Anopheles* followed by ponds and swamps. The great majority of larvae inhabiting these habitats were *An. gambiae,* followed by *An. cinereus* and *An. chrysti*. In Ethiopia, *Anopheles arabiensis*, a sibling species in the *An. gambiae* complex, is widely distributed and remains a major malaria vector throughout the country. Previous studies reported occurrence of *An. arabiensis* and other species of *Anopheles* larvae along streams in a central highland area [18] and in the middle course of the rift valley [20] of Ethiopia. Studies from other east African countries also support this observation [21–23].

Abundance of *An. gambiae* larvae along streams and also in ponds and swamps during dry seasons entails a strengthened vector control strategy in the habitats against aquatic stages of anophelines during the seasons. Management of the streams during dry seasons can reduce the vector populations significantly as the aquatic stages (eggs, larvae and pupae) of mosquitoes remain confined within relatively small habitats and cannot readily escape control measures [6, 24–26]. Disruption of the aquatic stages of mosquitoes during the dry seasons would decrease the size of an already small population, and may delay an explosive build-up of the vector populations towards the end of dry seasons, during the onset of the rainy seasons and following the rains in inland villages [10, 19, 24, 27]. It can also serve as an alternative control strategy to malaria transmitting vectors that are resistant to the available insecticide based control tools (such as LLINs and IRS) [28–30] and hence can be highly effective in controlling malaria transmission in Jabi Tehnan district if used and also integrated in the current malaria vector control package [4]. As the streams are used for washing clothes and as sources of drinking water for cattle (personal observation during the study), chemical based larval intervention cannot be employed. Instead, temporary and scheduled manipulation of the habitats such as straightening, flashing and changing their course can be employed.

Indoor spray catches revealed occurrence of adults of *An. gambiae*, *An. chrysti*, *An. demeilloni*, *An. cinereus* and *An. coustani* in a decreasing order of density. *Anopheles gambiae,* which comprises *An. arabiensis* and *Anopheles amharicus* in Ethiopia, was the most frequently caught mosquito. Most previous studies reported *An. arabiensis* from different parts of the country and is the main vector of malaria, while *An. amharicus* (formerly *Anopheles quadriannulatus* species B) was described to occur in the country [30–33]. *An. arabiensis* is most frequently reported to occur in dry seasons than any other species in the *An. gambiae* [18, 25, 34]. Hence, the *An. gambiae* catches can presumably be considered as *An. arabiensis* and could be considered the primary vector responsible for the transmission of *Plasmodium falciparum* and *Plasmodium vivax* malaria in the area during the seasons [13, 14]. However, it remains important to incriminate *An. cinereus*, *An. chrysti*, *An. demeilloni* and *An. coustani* for their vectorial role in the area.

The houses from where adult mosquitoes surveyed, both located within 1 km radius from a nearby permanent breeding habitat, were observed to have similar density of indoor resting *An. gambiae*. This could partly be explained by the similar housing conditions and the indoor occurrence of the blood meal sources readily in every house during the night hours. However, this observation differs from previous studies in which case the indoor density of *Anopheles* varied significantly among houses within the same village [12, 25, 35, 36]. The indoor occurrence of the vectors could maintain transmission of the malaria during the dry season and serve as sources for increased transmission intensity in the villages at the time of vector population built up [27]. In the area, the domestic animals and humans share the same house in the night creating favourable blood meal source for the mosquitoes. Species of the *An. gambiae* complex feed during night hours and *An. arabiensis* a species in the complex feeds on humans and cattle alternatively [17]. This may suggest the possibility of indoor feeding and resting activity of the *An. gambiae* in Jabi Tehnan District.

The occurrence of adult anopheline mosquitoes inside residential houses indicates the need for proper utilization of existing malaria vector control tools namely the insecticide treated mosquito nets and indoor residual spraying. The use of improved housing conditions remains a top priority in view of the increasing abundance of insecticide resistant malaria vectors and also sustaining the low level transmission of malaria in the district.

It was not possible to carry out immature and adult *Anopheles* survey during every month of the year and hence describe the peak and low *Anopheles* density season(s). As a result it was not possible to clearly describe occurrence of the mosquitoes and the respective prolific breeding habitats during other seasons. It was not also possible to test the sporozoite infection status of the mosquitoes due to the shortage of reagents.

## Conclusion

The streams were prolific breeding habitats of *Anopheles* followed by ponds and swamps during dry seasons in Jiga Yehlmidar, Mender Meter and Wongie Berkegn villages, Jabi Tehnan District, West Gojjam Zone, Ethiopia. A high population of indoor resting *Anopheles gambiae*, followed by *An. cinereus*, *An. chrysti*, *An. demeilloni* and *An. coustani* were caught in the villages during the seasons. This implies the need to carry out management of breeding habitats, improved housing and scaled-up use of insecticide (LLINs and IRS) based adult vector control tools, during dry seasons, to decrease the population of malaria transmitting *Anopheles* mosquitoes in the villages.
